# Prediction of Pancreatic Cancer in Diabetes Patients with Worsening Glycemic Control

**DOI:** 10.1158/1055-9965.EPI-21-0712

**Published:** 2021-11-02

**Authors:** Christie Y. Jeon, Sungjin Kim, Yu-Chen Lin, Harvey A. Risch, Mark O. Goodarzi, Teryl K. Nuckols, Stephen J. Freedland, Stephen J. Pandol, Joseph R. Pisegna

**Affiliations:** 1 Cedars-Sinai Cancer, Cedars-Sinai Medical Center, Los Angeles, California.; 2 Department of Epidemiology, UCLA Fielding School of Public Health, Los Angeles, California.; 3 Department of Epidemiology, Yale School of Public Health, Los Angeles, California.; 4 Division of Endocrinology, Diabetes and Metabolism, Cedars-Sinai Medical Center, Los Angeles, California.; 5 Division of General Internal Medicine, Cedars-Sinai Medical Center, Los Angeles, California.; 6 Department of Surgery, Cedars-Sinai Medical Center, Los Angeles, California.; 7 Section of Urology, Durham VA Medical Center, Durham, North Carolina.; 8 Karsh Division of Gastroenterology and Hepatology, Cedars-Sinai Medical Center, Los Angeles, California.; 9 Veterans Affairs Greater Los Angeles Healthcare System, Los Angeles, California.

## Abstract

**Background:**

Worsening glycemic control indicates elevated risk of pancreatic ductal adenocarcinoma (PDAC). We developed prediction models for PDAC among those with worsening glycemic control after diabetes diagnosis.

**Methods:**

In 2000–2016 records within the Veterans Affairs Health System (VA), we identified three cohorts with progression of diabetes: (i) insulin initiation (*n* = 449,685), (ii) initiation of combination oral hypoglycemic medication (*n* = 414,460), and (iii) hemoglobin A1c (HbA1c) ≥8% with ≥Δ1% within 15 months (*n* = 593,401). We computed 12-, 36-, and 60-month incidence of PDAC and developed prediction models separately for males and females, with consideration of >30 demographic, behavioral, clinical, and laboratory variables. Models were selected to optimize Akaike's Information Criterion, and performance for predicting 12-, 36-, and 60-month incident PDAC was evaluated by bootstrap.

**Results:**

Incidence of PDAC was highest for insulin initiators and greater in males than in females. Optimism-corrected c-indices of the models for predicting 36-month incidence of PDAC in the male population were: (i) 0.72, (ii) 0.70, and (iii) 0.71, respectively. Models performed better for predicting 12-month incident PDAC [c-index (i) 0.78, (ii) 0.73, (iii) 0.76 for males], and worse for predicting 60-month incident PDAC [c-index (i) 0.69, (ii) 0.67, (iii) 0.68 for males]. Model performance was lower among females. For subjects whose model-predicted 36-month PDAC risks were ≥1%, the observed incidences were (i) 1.9%, (ii) 2.2%, and (iii) 1.8%.

**Conclusions:**

Sex-specific models for PDAC can estimate risk of PDAC at the time of progression of diabetes.

**Impact:**

Our models can identify diabetes patients who would benefit from PDAC screening.

## Introduction

Only 10% of patients with pancreatic ductal adenocarcinoma (PDAC) survive beyond five years (1). Fewer than 25% of PDAC cases in the United States are diagnosed at a resectable stage (1, 2). Currently, no early detection strategy exists for the general population, and biomarkers such as CA19-9 and CA-125 have not translated to meaningful gains in early detection due to insufficient diagnostic accuracy (3–5).

Many nonspecific symptoms and comorbidities commonly develop in parallel with PDAC development and are likely indicators of early disease (6–11). A notable PDAC indicator is new-onset diabetes, which is present in 15% to 35% of PDAC patients (6, 10, 12, 13) and is associated with a 4-fold increased risk of PDAC (6–8). Initiation of insulin is even more strongly associated with risk of PDAC, with a relative risk of 5.6, and 45% of PDAC cases with diabetes have been treated with insulin (6). Although new-onset diabetes is a well-recognized risk factor for PDAC, worsening glycemic control among people with a known diagnosis of diabetes has not received similar attention as a PDAC risk factor. Worsening glucose control could prompt a regimen change, such as adding a second or third hypoglycemic agent or starting insulin, at which the risk of PDAC may also be assessed.

A number of prospective PDAC prediction models have been reported for new-onset diabetes in the literature (14–16), but none has focused on progression of diabetes. In our study, we built a refined prediction model to estimate the risk of PDAC from the time of progression of diabetes, considering predictors from models for new-onset diabetes, utilizing longitudinal electronic medical records (EMR) from the Veterans Affairs Health System (VA; ref. 17). We also incorporated duration of clinical risk factors, such as use of proton-pump inhibitor (PPI), which have shown to improve model performance (18). We also developed sex-specific models, given that risk of PDAC is higher in males than in females. VA was specifically chosen given that veterans represent a large proportion of the U.S. population and that the VA electronic health system is one of the oldest nationwide EMR in the United States.

## Materials and Methods

### Data source

Our study data originate from the Department of Veterans Affairs Corporate Data Warehouse (CDW), a nationwide VA database that collates metadata on electronic health and administrative information on patients at all regional VA healthcare centers. The CDW contains demographic data, inpatient and outpatient clinical data, laboratory test results, and diagnostic and procedure codes.

### Cohort definitions

To select persons with progression of diabetes after diagnosis of diabetes, we first identified a population ages 50 or above with diabetes by diagnostic codes, laboratory results of glucose and HbA1c, and pharmaceutical records using definitions for diabetes previously used in the VA population (19): (i) at least two outpatient visits with a VA primary care provider with ICD-9 code of 250.xx, or ICD-10 code of E11.x, and (ii) HbA1c of ≥6.5%, fasting glucose of ≥126 mg/dL, or random blood glucose of ≥200 mg/dL. Laboratory tests on blood collected during hospitalization or emergency department visit were excluded, given that acute conditions could temporarily elevate glucose levels (20). Among this pool of diabetes patients, we identified three nonindependent populations representing different stages of diabetes: (i) initiation of insulin, (ii) initiation of combination (two or more) oral hypoglycemic treatment from monotherapy, or (iii) ≥1% increase in hemoglobin A1c (HbA1c) with the last HbA1c measuring ≥8%. These were chosen in consultation with expert endocrinologists as populations in whom a prediction model for PDAC would be useful for differentiating the origin of worsening glycemic control. Cohort entry was defined as the date of first prescription for insulin in population (i), as the date of simultaneous prescription for two oral hypoglycemic drugs in population (ii), and as the first date of when HbA1c measured ≥8% with a prior HbA1c value within 15 months that was lower by 1% or more, in population (iii). We excluded from these cohorts patients with apparent onset of progression within 90 days after first-ever evidence of diabetes, because although they were newly discovered to have diabetes, they likely had diabetes for some time and the progression timing is unclear. Sex-specific models were developed in each of the non-mutually exclusive population with diabetes. Patients who experienced more than one definition of progression were allowed to contribute to multiple cohorts. As a comparison, we also estimated the age-adjusted risk of PDAC among patients with diabetes who did not meet any of the above definition of progression (nonprogressors). Of note, the focus of our study was to build prediction models for PDAC among diabetes patients with progression, and not to estimate the relative risks of PDAC attributable to progression of diabetes.

### Definition of incident PDAC

We ascertained incident PDAC through cancer registries in the VA, through EMR, and through Medicare claims. We first identified cases with primary pancreatic adenocarcinoma as recorded in the VA Central Cancer Registry. Because the VA Cancer Registry does not capture all cancer cases that are diagnosed within the VA (21), we additionally identified persons who had at least two encounters at the VA with an ICD diagnosis of PDAC (ICD-9, 157.0, 157.1, 157.2, 157.3, 157.9, or ICD-10 diagnoses of C25.0, C25.1, C25.2, C25.3, C25.8, C25.9). Finally, patients with at least two independent Medicare claims for PDAC (same ICD codes as above) were added, given that VA patients may have been diagnosed with PDAC outside the VA setting. Patients who did not develop PDAC were censored at the last known vital status date as recorded in the VA CDW, or December 31, 2017.

### Covariates

We extracted data on the following risk factors in persons with progressing diabetes. All covariate data were assessed based on records available on or prior to the index date in each cohort. *Demographic data*: Age at the time of progression of diabetes in each respective cohort, sex, race (five census categories), and Hispanic ethnicity. *Smoking* status was categorized as never, former, or current smoker, assessed up to the respective time of progression of diabetes. *Alcohol consumption*: Heavy drinking was determined by the highest AUDIT-C score prior to progression of diabetes. AUDIT-C is a validated screening tool for alcohol use disorders (22), utilized throughout the VA since 2008. A score of ≥4 for men and ≥3 for women is indicative of hazardous drinking. *Comorbidity*: We identified patients with acute or chronic pancreatitis, dyspepsia/gastritis/peptic ulcer disease, abdominal pain, nonalcoholic fatty liver disease (NAFLD), heart disease, jaundice, or alcoholism by ICD-9/10 codes. Patients with a history of both acute and chronic pancreatitis were classified as having chronic pancreatitis. *Medications:* Because the use of PPIs can indicate upper abdominal discomfort related to the pancreas, we extracted data on prescriptions for the following commonly used PPIs: pantoprazole, omeprazole, esomeprazole, lansoprazole, and rabeprazole. Given that PDAC risk varies by exposure to metabolic agents such as statins and metformin (23–25), we extracted data on prescriptions for statins and diabetes medications including biguanides, sulfonylureas, thiazolidinediones, alpha-glucosidase inhibitors, DPP-4 inhibitors, and GLP-1 receptor agonists. *Metabolic parameters:* Obesity and recent weight loss are risk factors for PDAC (15, 26). We therefore extracted data on the highest weight and height ever recorded on a patient prior to cohort entry to determine the peak BMI. Weight values in the range of 75 to 500 lbs and height values in the range of 48 to 84 inches were considered (27). Change in weight was assessed by percentage of loss in weight compared with prior weight measured ∼12 months before, within a 3- to 15-month window. We also extracted data on laboratory tests: HbA1c, creatinine, cholesterol, bilirubin, hemoglobin, red blood cell (RBC), which have been significantly associated with PDAC development in a previous study (14). Laboratory test values most proximal to the “onset” of progression of diabetes within 12 months prior to the index date were entered in the model. Because physiologic changes could indicate elevated risk of PDAC, for each laboratory parameter, we computed the percentage change in lab values from a test value closest to 12 months (within a 3–15-month window) prior to the last test. Patients with incomplete data on the continuous parameters were excluded. These comprised less than 10% of the data.

### Statistical analysis

All analyses were performed separately for each population (six populations in total from three diabetes cohorts stratified by sex, male/female). Patient characteristics are presented as number of patients (%) or median (IQR, interquartile range) overall and stratified by incident PDAC. The primary outcome is incident PDAC defined as time from progression of diabetes to incident PDAC. Median follow-up time was calculated using the reverse Kaplan–Meier method (28). Age-adjusted cumulative incidences of PDAC were estimated standardized to age 60. Univariate and multivariable analyses were conducted to examine associations between incident PDAC and its potential predictors using Cox proportional hazards regression models (29). The proportional hazards assumption was assessed with scaled Schoenfeld residuals (30).

Because more recent diagnoses of comorbid conditions or prescription drugs are more strongly associated with incident PDAC than diagnoses or prescriptions made in the distant past (18), comorbid conditions (e.g., acute pancreatitis) and prescription drugs (e.g., statins) were modeled as exponential decay of the log hazard ratio according to the number of months in the past when the diagnosis or prescription occurred using the iterative linearization method (31). Each of the conditions or drugs was included in models as an interaction term in the form of *β_1 × I*[exp (*f*_0_)(*−β_2×t*)], where *β_1* is the parameter estimate of the diagnosis of the condition or prescription of drug, *I* denotes an indication of the diagnosis of the condition or prescription of drug (0 or 1), *β_2* is the parameter estimate of the time in the past before the diagnosis or prescription occurred, and *t* is months in the past before cohort entry date. Corresponding confidence intervals for the variables are presented as a function of time before cohort entry using the delta method according to estimated standard errors.

Model selection was performed using a stepwise variable selection procedure based on Akaike information criterion (AIC) considering all but duration (32). Duration variables were added to models regardless of their significance in relation to incident PDAC if corresponding diagnosis or prescription variables were retained in the model. In multivariable analyses, the possibility of collinearity was reduced through the careful initial assessment of correlations among study covariates.

The performance of multivariable models predicting incident PDAC was assessed with measures of discrimination and calibration (33). Discriminative ability of models was measured at 12, 36, and 60 months using time-dependent area under the receiver operating characteristics curves (c-statistic) with the use of cumulative sensitivity/dynamic specificity (34). Calibration of the prediction models was evaluated with calibration slope. Internal validation of the models was performed by estimating and correcting possible overfitting and optimism in the model performance estimates using the bootstrap method with 100 to 300 replicates (35), which provides stable estimates with low bias than split-sample procedure (36, 37). Estimated optimism-corrected performance measures were reported. The predicted risks for incident PDAC in 12, 36, and 60 months were estimated for each study population. Then, sensitivity, specificity, and positive predictive value (PPV) were estimated at predicted risk thresholds of 0.5%, 1%, and 2% (test positive if the estimated predicted risk ≥ threshold of interest; negative, otherwise) along with 95% exact confidence intervals. Sensitivity and specificity of the models would vary by thresholds, as higher thresholds would be more specific at the cost of lower sensitivity, and lower thresholds would be more sensitive at the cost of reduced specificity.

All analyses were performed using SAS 9.4 (SAS Institute, Inc.) and R package version 4.0.2 (R Foundation) with two-sided tests at a significant level of 0.05. The IRB of VA Greater Los Angeles (Pro#1615788) and Cedars-Sinai Medical Center (Pro#51233) approved of the study.

### Data availability statement

Individual-level data are not available for the public, per VA data use guidelines for research. Aggregate-level data and model specifications are reported in the manuscript.

## Results

### Study population

Of 1,546,101 patients with diabetes, 799,529 experienced one of the three definitions of progression: (i) 449,685 patients with new insulin treatment (438,816 male; 10,869 female); (ii) 414,460 with new combination oral hypoglycemic treatment (404,858 male; 9,602 female); (iii) 593,401 patients with ≥1% increase in HbA1c, with the recent HbA1c ≥8% (579,384 male; 14,017 female). Of 12,412 PDAC cases identified among diabetes patients, 6,300 cases (51%) occurred after progression of diabetes. In each cohort, (i) 3,675, (ii) 3,150, (iii) 4,606 male patients and (i) 54, (ii) 48, (iii) 66 female patients developed PDAC. The distribution of the three populations and their overlap, and distribution of the PDAC cases between the three cohorts are presented in Supplementary Figs. S1 and S2. PDAC was identified through the VA Central Cancer Registry in 45% to 46% of the PDAC patients, through EMR in 30% to 31% of the patients, and through Medicare claims in 23% to 24% of the patients in the three cohorts. Median follow-up was 73.5 months in cohort (i), 93.8 months in cohort (ii), and 77.7 months in cohort (iii). Tables 1 and 2 describe the distribution of selected model parameters in male and female populations with diabetes progression, respectively. Among males, median age at time of progression of those who remained PDAC-free ranged 64.0 to 64.7 years, and median age of those who developed PDAC ranged 65.2 to 66.2. Black and Hispanic patients comprised 16% to 18% and 6% to 7% of the male populations, respectively. Among females, median age at time of progression of those who remained PDAC-free ranged 58.3 to 58.8 years, and median age of those who developed PDAC ranged 60.5 to 61.2. Black and Hispanic patients comprised 26%, and 5% to 6% of the female populations, respectively.

**Table 1.** Distribution of model parameters in three populations with diabetes progression, male.

		Patients initiating insulin for diabetes		Patients initiating combination oral hypoglycemic treatment for diabetes		Population with ≥1% increase in HbA1c over 8%	
		*n* (column %) or median (IQR)		*n* (column %) or median (IQR)		*n* (column %) or median (IQR)	
Variable	Category	Pancreatic cancer-free (*n* = 435,141)	Pancreatic cancer (*n* = 3,675)	Pancreatic cancer-free (*n* = 401,708)	Pancreatic Cancer (*n* = 3,150)	Pancreatic cancer-free (*n* = 574,778)	Pancreatic cancer (*n* = 4,606)
Age (years)	(Median, IQR)	64.7 (59.2–71.7)	65.8 (60.6–72.9)	64.0 (58.5–70.2)	65.2 (59.7–71.8)	64.5 (58.8–71.2)	66.2 (60.4–72.9)
Race	White	346,375 (79.6)	2,943 (80.08)	325,602 (81.05)	2,548 (80.89)	454,708 (79.11)	3,681 (79.92)
	Black	75,714 (17.4)	633 (17.22)	63,432 (15.79)	525 (16.67)	102,116 (17.77)	801 (17.39)
	Asian	1,831 (0.42)	14 (0.38)	2,186 (0.54)	10 (0.32)	2,662 (0.46)	12 (0.26)
	Other	11,221 (2.58)	85 (2.31)	10,488 (2.61)	67 (2.13)	15,292 (2.66)	112 (2.43)
Hispanic ethnicity		26,117 (6)	195 (5.31)	25,287 (6.29)	163 (5.17)	39,850 (6.93)	270 (5.86)
Smoking status	Current smoker	116,690 (26.82)	1,185 (32.24)	107,942 (26.87)	939 (29.81)	158,616 (27.6)	1,415 (30.72)
	Former smoker	179,743 (41.31)	1,424 (38.75)	150,331 (37.42)	1,087 (34.51)	231,976 (40.36)	1,763 (38.28)
	Never smoker	82,165 (18.88)	601 (16.35)	78,240 (19.48)	528 (16.76)	111,149 (19.34)	792 (17.19)
	Missing/unknown	56,543 (12.99)	465 (12.65)	65,195 (16.23)	596 (18.92)	73,037 (12.71)	636 (13.81)
Heavy drinking	No	190,773 (43.84)	1,414 (38.48)	130,114 (32.39)	753 (23.9)	232,814 (40.51)	1,561 (33.89)
	Yes	34,689 (7.97)	289 (7.86)	28,959 (7.21)	161 (5.11)	48,757 (8.48)	373 (8.1)
	Unknown/missing	209,679 (48.19)	1,972 (53.66)	242,635 (60.4)	2,236 (70.98)	293,207 (51.01)	2,672 (58.01)
Acute pancreatitis		9,884 (2.27)	202 (5.5)	5,374 (1.34)	89 (2.83)	11,132 (1.94)	201 (4.36)
Chronic pancreatitis		4,822 (1.11)	98 (2.67)	2,482 (0.62)	44 (1.4)	5,843 (1.02)	107 (2.32)
Abdominal pain		68,560 (15.76)	760 (20.68)	51,734 (12.88)	471 (14.95)	89,424 (15.56)	844 (18.32)
NAFLD		15,107 (3.47)	159 (4.33)	11,005 (2.74)	81 (2.57)	18,540 (3.23)	150 (3.26)
Heart disease		194,390 (44.67)	1,586 (43.16)	148,608 (36.99)	1,230 (39.05)	242,062 (42.11)	1915 (41.58)
Jaundice		1,961 (0.45)	68 (1.85)	1,224 (0.3)	16 (0.51)	2,472 (0.43)	42 (0.91)
Alcoholism		141,868 (32.6)	1,376 (37.44)	123,148 (30.66)	1,018 (32.32)	190,339 (33.12)	1,680 (36.47)
Cirrhosis		9,237 (2.12)	77 (2.1)	4,562 (1.14)	46 (1.46)	10,575 (1.84)	97 (2.11)
Back pain		71,453 (16.42)	629 (17.12)	58,918 (14.67)	458 (14.54)	95,176 (16.56)	790 (17.15)
Proton-pump inhibitor		182,794 (42.01)	1,602 (43.59)	140,139 (34.89)	1,110 (35.24)	230,024 (40.02)	1,872 (40.64)
Biguanides		331,997 (76.3)	2,898 (78.86)	383,810 (95.54)	3,006 (95.43)	417,996 (72.72)	3,316 (71.99)
DPP-IV inhibitors		10,571 (2.43)	65 (1.77)	8,493 (2.11)	37 (1.17)	5,472 (0.95)	25 (0.54)
Weight (lbs)		216 (188.2–248.6)	207 (179.5–239.6)	218 (192–249)	213.05 (187–244)	218.8 (191–250.8)	211 (184.7–242.6)
% weight change		−0.43 (−3.43 to 2.26)	−1.68 (−5.78 to 1.43)	0 (−2.6 to 2.36)	−0.54 (−3.45 to 2.1)	0.18 (−2.48 to 3.06)	−0.49 (−3.57 to 2.45)
Peak BMI		32.99 (29.33–37.43)	32.2 (28.76–36.58)	32.82 (29.33–37.03)	32.29 (28.76–36.24)	32.99 (29.42–37.33)	32.21 (28.83–36.4)
HbA1C (%)		8.7 (7.6–10.2)	8.9 (7.8–10.4)	7.8 (7.1–9)	7.8 (7.1–9)	9 (8.4–10)	9 (8.4–10)
HbA1c change (%)		0.2 (−0.2 to 1.4)	0.5 (−0.1 to 1.8)	0 (−0.5 to 0.7)	0 (−0.4 to 0.8)	1.4 (1–2.1)	1.5 (1–2.2)
Creatinine (mg/dL)		1.1 (0.9–1.4)	1.07 (0.9–1.3)	0 (−9.09 to 10)	0 (−9.09 to 10)	1.1 (0.9–1.3)	1.07 (0.9–1.28)
Cholesterol (mg/dL)		163 (138–193)	159 (135–189)	166 (142–195)	163 (140–192)	166 (142–196)	162 (138–191)
Bilirubin (mg/dL)		0.6 (0.4–0.8)	0.6 (0.4–0.8)	0.6 (0.4–0.8)	0.6 (0.4–0.8)	0.6 (0.4–0.8)	0.6 (0.4–0.8)
% Bilirubin change		0 (−20 to 25)	0 (−18.18 to 28.57)	0 (−20 to 20)	0 (−20 to 20)	0 (−20 to 25)	0 (−20 to 25)
RBC (M/cmm)		4.65 (4.25–5.01)	4.62 (4.24–4.98)	4.78 (4.45–5.09)	4.74 (4.42–5.06)	4.74 (4.39–5.07)	4.71 (4.36–5.03)
% RBC change		0 (−5.08 to 2.87)	0 (−5.6 to 3.03)	0 (−4.05 to 2.21)	0 (−4.44 to 1.81)	0 (−3.94 to 4.18)	0 (−4.29 to 3.8)

Abbreviations: IQR, interquartile range; *n*, number of patients.

**Table 2.** Distribution of model parameters in three populations with diabetes progression, female.

		Patients initiating insulin for diabetes		Patients initiating combination oral hypoglycemic treatment for diabetes		Population with ≥1% increase in HbA1c over 8%	
		*n* (column %) or median (IQR)		*n* (column %) or median (IQR)		*n* (column %) or median (IQR)	
Variable	Category	Pancreatic cancer-free (*n* = 10,815)	Pancreatic cancer (*n* = 54)	Pancreatic cancer-free (*n* = 9,554)	Pancreatic cancer (*n* = 48)	Pancreatic cancer-free (*n* = 13,951)	Pancreatic cancer (*n* = 66)
Age (years)		58.8 (54.6–64.2)	60.5 (58.3–65.0)	58.3 (54.3–63.6)	60.7 (56.9–64.0)	58.7 (54.4–64.2)	61.2 (58.5–67.1)
Race	White	7,555 (69.86)	38 (70.37)	6,693 (70.05)	36 (75)	9,688 (69.44)	46 (69.7)
	Black	2,839 (26.25)	15 (27.78)	2,480 (25.96)	11 (22.92)	3,693 (26.47)	20 (30.3)
	Asian	57 (0.53)	0 (0)	62 (0.65)	0 (0)	93 (0.67)	0 (0)
	Other	364 (3.37)	1 (1.85)	319 (3.34)	1 (2.08)	477 (3.42)	0 (0)
Hispanic Ethnicity		566 (5.23)	2 (3.7)	573 (6)	3 (6.25)	816 (5.85)	1 (1.52)
Smoking status	Current smoker	2,974 (27.5)	21 (38.89)	2,628 (27.51)	12 (25)	3,895 (27.92)	17 (25.76)
	Former smoker	3,325 (30.74)	9 (16.67)	2,750 (28.78)	10 (20.83)	4,198 (30.09)	22 (33.33)
	Never smoker	3,544 (32.77)	16 (29.63)	3,201 (33.5)	17 (35.42)	4,744 (34)	20 (30.3)
	Missing/unknown	972 (8.99)	8 (14.81)	975 (10.21)	9 (18.75)	1114 (7.99)	7 (10.61)
Heavy drinking	No	5,839 (53.99)	22 (40.74)	4,182 (43.77)	10 (20.83)	7,145 (51.21)	27 (40.91)
	Yes	619 (5.72)	2 (3.7)	497 (5.2)	2 (4.17)	845 (6.06)	2 (3.03)
	Unknown/missing	4,357 (40.29)	30 (55.56)	4,875 (51.03)	36 (75)	5,961 (42.73)	37 (56.06)
Acute pancreatitis		251 (2.32)	3 (5.56)	141 (1.48)	2 (4.17)	280 (2.01)	2 (3.03)
Chronic pancreatitis		110 (1.02)	3 (5.56)	60 (0.63)	0 (0)	124 (0.89)	1 (1.52)
Abdominal pain		3,306 (30.57)	20 (37.04)	2,673 (27.98)	14 (29.17)	4,297 (30.8)	23 (34.85)
NAFLD		683 (6.32)	6 (11.11)	521 (5.45)	3 (6.25)	849 (6.09)	6 (9.09)
Heart disease		2,975 (27.51)	14 (25.93)	2,221 (23.25)	18 (37.5)	3,670 (26.31)	22 (33.33)
Jaundice		23 (0.21)	1 (1.85)	13 (0.14)	0 (0)	29 (0.21)	1 (1.52)
Alcoholism		3,439 (31.8)	20 (37.04)	2,844 (29.77)	17 (35.42)	4,467 (32.02)	20 (30.3)
Cirrhosis		183 (1.69)	1 (1.85)	90 (0.94)	0 (0)	215 (1.54)	2 (3.03)
Diabetic neuropathy		2,336 (21.6)	9 (16.67)	1,642 (17.19)	7 (14.58)	2,958 (21.2)	16 (24.24)
Diabetic nephropathy		595 (5.5)	2 (3.7)	357 (3.74)	0 (0)	716 (5.13)	2 (3.03)
Back pain		2,922 (27.02)	17 (31.48)	2,382 (24.93)	10 (20.83)	3,840 (27.52)	20 (30.3)
Proton-pump inhibitor		5,671 (52.44)	31 (57.41)	4,554 (47.67)	25 (52.08)	7,224 (51.78)	31 (46.97)
Biguanides		8,863 (81.95)	45 (83.33)	9,344 (97.8)	48 (100)	11,060 (79.28)	53 (80.3)
DPP-IV inhibitors		347 (3.21)	0 (0)	344 (3.6)	0 (0)	188 (1.35)	0 (0)
Weight (lbs)		196.2 (169–227)	180.6 (154–204.9)	197.7 (171–228)	174.65 (153–218.9)	199 (172–229.9)	193.2 (166.3–218.2)
% weight change		−0.34 (−3.55 to 2.76)	−2.35 (−7.83 to 2.96)	−0.3 (−3.31–2.6)	−0.54 (−3.25 to 2.4)	0.65 (−2.41 to 4.08)	1.33 (−2.76 to 4.85)
Peak BMI		35.34 (30.98–40.45)	33.4 (30.13–38.31)	35.34 (30.95–40.35)	32.86 (28.96–39.08)	35.34 (31–40.33)	34.18 (31.23–41.09)
HbA1c (%)		9 (7.8–10.6)	8.65 (7.7–10.5)	8 (7.2–9.3)	7.65 (7.2–8.35)	9.1 (8.4–10.2)	9 (8.3–10)
HbA1c change (%)		0.3 (−0.2 to 1.7)	0.3 (0–2.4)	0 (−0.5 to 0.9)	0 (−0.95 to 0.4)	1.5 (1–2.2)	1.5 (0.8–2)
Creatinine (mg/dL)		0.8 (0.7–1)	0.9 (0.6–1.1)	0.8 (0.7–0.96)	0.8 (0.72–0.99)	0.8 (0.7–1)	0.9 (0.71–1.1)
Cholesterol (mg/dL)		183 (155–218)	185 (152–207)	182 (157–217)	168.5 (155–205.5)	185 (159–220)	181.5 (155–212)
Bilirubin (mg/dL)		0.5 (0.34–0.7)	0.4 (0.3–0.6)	0.5 (0.34–0.7)	0.5 (0.4–0.6)	0.5 (0.39–0.7)	0.5 (0.34–0.65)
% Bilirubin change		0 (−20 to 25)	0 (−24 to 50)	0 (−20 to 25)	0 (−15.43 to 30.95)	0 (−20 to 33.33)	0 (−27.27 to 33.33)
RBC (M/cmm)		4.51 (4.18–4.82)	4.39 (4.15–4.69)	4.56 (4.26–4.85)	4.45 (4.29–4.74)	4.54 (4.25–4.84)	4.5 (4.03–4.73)
% RBC change		0 (−4.31 to 3.86)	0 (−4.36 to 4.37)	0 (−3.95 to 3.1)	0 (−5.24 to 2.25)	0 (−3.29 to 4.95)	0 (−5.42 to 5.21)

Abbreviations: IQR, interquartile range; *n*, number of patients.

A unique feature of our models is the inclusion of time since onset of clinical predictors of PDAC. We summarized in Supplementary Table S1, the number of days from first indication of the clinical predictor to the respective onset of progression of diabetes. Time since onset of predictor was shorter in patients who developed PDAC as opposed to those who remained PDAC-free for several indicators: acute pancreatitis, chronic pancreatitis, abdominal pain, and jaundice.

### Incidence of PDAC

**Figure 1.**
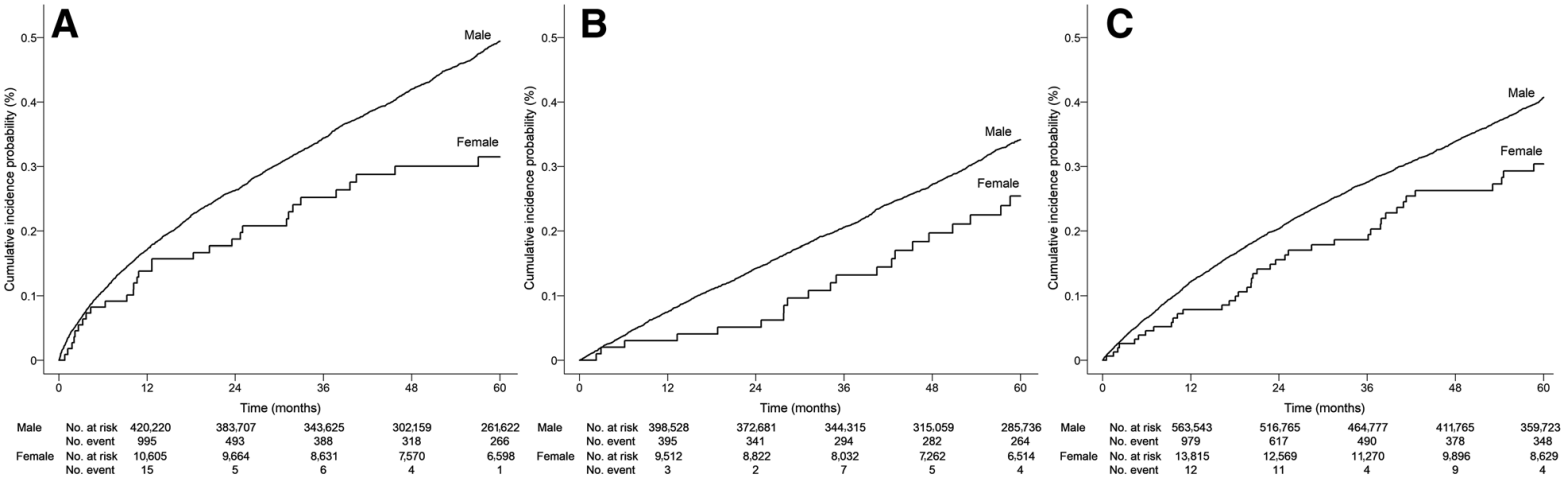
Age-adjusted cumulative incidence estimates of pancreatic cancer in patients aged 60 years with progression of diabetes. **A,** Diabetes patients with insulin initiation; **B,** Diabetes patients initiating combination oral hypoglycemic treatment; **C,** Diabetes patients with ≥1% increase in A1c over 8%.

Age-adjusted cumulative incidence of PDAC at 12, 24, 36, 48, and 60 months standardized to 60 years of age at progression of diabetes is presented in Supplementary Table S2 and illustrated in Fig. 1. Incidence of PDAC was highest for the insulin-initiating male cohort, rising more steeply in the first 12 months to an incidence of 0.18% [180 per 100,000 person-years (p-y)] and reached 0.52% over 60 months from the time of the first prescription for insulin. The incidence was lower in female patients initiating insulin, with 12- and 60-month PDAC incidence of 0.13% and 0.30%, respectively. The incidence of PDAC in diabetes patients with increasing HbA1c also rose more steeply in the first 12 months to an incidence of 0.13% in males and 0.08% in females. The 60-month incidence of PDAC in the elevated A1c cohort was 0.44% in males and 0.31% in females. In comparison with insulin users and those with increasing HbA1c, the incidence of PDAC in diabetes patients initiating combination oral hypoglycemic treatment or those who did not meet any of the progression criteria was lower (Supplementary Table S2).

### Multivariable model and independent risk factors of PDAC

Results of the selected multivariable model are presented in Table 3. In all models, increase in age was associated with PDAC. In models developed among men, Hispanic ethnicity was inversely associated with PDAC, whereas current smoking was positively associated with PDAC risk. Acute pancreatitis, abdominal pain, jaundice, and alcoholism were uniformly selected in all three models among male diabetes patients, with current abdominal pain and current jaundice showing stronger associations with PDAC than that diagnosed in more distant past. Among continuous variables, 20% weight increase was consistently and strongly associated with lower risk of PDAC (HR = 0.56–0.70), as was higher levels of HbA1c (2%–5% increased risk of PDAC per 1% higher level of the most recent HbA1c). Higher levels of creatinine and cholesterol were associated with decreased risk of PDAC. Chronic pancreatitis was not selected in the model for cohort (i), but selected in models for cohorts (ii) and (iii). Weight was associated with decreased risk of PDAC independent of weight change in cohorts (i), and (iii) and not selected in cohort (ii). Change in HbA1c associated with PDAC in cohorts (i) and (iii), independent of the most recent HbA1c levels. Similarly, percent change in bilirubin was associated with PDAC risk in cohorts (i) and (iii). Higher level of RBC was associated with reduced risk of PDAC in models for cohorts (ii) and (iii).

**Table 3.** Multivariable associations with pancreatic cancer in three populations with diabetes progression.

		Initiating insulin for diabetes		Initiating combination oral hypoglycemic treatment for diabetes		≥1% increase in HbA1c over 8%	
		HR (95% CI)		HR (95% CI)		HR (95% CI)	
Variable	Category or unit for HR estimation	Male	Female	Male	Female	Male	Female
Age (years)	1 unit increase	1.04 (1.04–1.05)	1.04 (1.00–1.07)	1.04 (1.03–1.04)	1.02 (0.99–1.05)	1.04 (1.04–1.05)	1.04 (1.01–1.06)
Race (Ref: White)	Black	NS	NS	1.10 (0.99–1.21)	NS	NS	NS
	Asian			0.68 (0.37–1.27)			
	Other			0.85 (0.66–1.08)			
Hispanic ethnicity (Ref: Non-Hispanic/Latino)	Hispanic or Latino	0.78 (0.67–0.90)	NS	0.79 (0.68–0.93)	NS	0.76 (0.67–0.86)	NS
	Unknown	1.49 (1.22–1.81)		1.27 (1.00–1.60)		1.50 (1.26–1.79)	
Smoking status (Ref: Never smoker)	Current smoker	1.33 (1.19–1.49)	NS	1.34 (1.18–1.51)	NS	1.21 (1.09–1.33)	NS
	Former smoker	1.05 (0.95–1.15)		1.08 (0.97–1.20)		1.03 (0.95–1.13)	
	Missing/unknown	0.93 (0.82–1.06)		1.05 (0.94–1.19)		1.00 (0.90–1.12)	
Heavy drinking (Ref: No)	Yes	1.08 (0.95–1.23)	NS	1.00 (0.84–1.19)	NS	1.16 (1.03–1.30)	NS
	Unknown/missing	0.84 (0.78–0.91)		0.76 (0.69–0.84)		0.76 (0.71–0.82)	
Acute pancreatitis	Current	7.26 (5.92–8.91)	NS	5.69 (4.03–8.04)	NS	13.49 (10.45–17.4)	NS
	3 years in the past	6.63 (5.57–7.89)		5.94 (4.53–7.79)		6.41 (4.92–8.35)	
Chronic pancreatitis	Current	NS	496.7 (108.6–2,271.3)	4.91 (2.76–8.73)	NS	1.96 (1.55–2.48)	NS
	3 years in the past		62.78 (2.06–1913.03)	2.78 (1.62–4.76)		2.43 (1.99–2.98)	
Abdominal pain	Current	3.02 (2.64–3.45)	NS	3.15 (2.64–3.76)	NS	2.26 (1.99–3.57)	NS
	3 years in the past	2.35 (2.13–2.60)		2.14 (1.84–2.48)		1.84 (1.67–2.02)	
NAFLD	Current	1.68 (1.34–2.10)	20.58 (5.74–73.74)	NS	NS	NS	14.46 (4.03–51.87)
	3 years in the past	1.72 (1.45–2.04)	13.42 (4.14–43.48)				12.36 (4.76–32.12)
Heart disease	Current	NS	NS	NS	8.91 (3.94–20.15)	NS	NS
	3 years in the past				6.76 (3.28–13.9)		
Jaundice	Current	207.9 (140.3–308.2)	NS	20.4 (7.79–53.44)	NS	22.48 (13.7–36.9)	NS
	3 years in the past	1.43 (0.95–2.15)		3.06 (0.98–9.49)		5.04 (2.62–9.72)	
Alcoholism	Current	1.46 (1.34–1.59)	NS	1.74 (1.55–1.96)	NS	1.64 (1.51–1.77)	NS
	3 years in the past	1.63 (1.51–1.76)		1.77 (1.61–1.94)		1.86 (1.74–1.99)	
Cirrhosis	Current	NS	NS	3.31 (2.09–5.24)	NS	NS	NS
	3 years in the past			3.08 (2.22–4.29)			
Back pain	Current	NS	NS	NS	NS	1.11 (1.06–1.17)	NS
	3 years in the past					1.21 (1.13–1.29)	
Neuropathy	Current	NS	NS	1.18 (1.07–1.31)	NS	NS	NS
	3 years in the past			1.27 (1.16–1.40)			
Proton-pump inhibitor	Current	NS	NS	1.34 (1.20–1.50)	NS	NS	NS
	3 years in the past			1.28 (1.17–1.41)			
Biguanides	Current	1.59 (1.44–1.76)	NS	NS	NS	NS	NS
	3 years in the past	1.43 (1.31–1.57)					
DPP-IV inhibitors	Current	NS	NS	2.64 (1.73–4.03)	NS	NS	NS
	3 years in the past			1.63 (0.42–6.29)			
Weight (lbs)	10-unit increase	0.96 (0.95–0.98)	NS	NS	0.93 (0.86–1.00)	0.98 (0.97–0.99)	NS
% weight change	20-unit increase	0.56 (0.50–0.62)	0.38 (0.19–0.76)	0.70 (0.61–0.79)	NS	0.60 (0.54–0.67)	NS
Peak BMI (kg/m^2^)	1 unit increase	1.02 (1.01–1.02)	NS	NS	NS	1.01 (1.00–1.02)	NS
HbA1C (%)	1 unit increase	1.04 (1.02–1.06)	NS	1.05 (1.03–1.08)	NS	1.02 (1.00–1.05)	NS
HbA1c change (%)	1 unit increase	1.03 (1.01–1.05)	NS	NS	NS	1.03 (1.00–1.05)	NS
Creatinine (mg/dL)	0.1 unit increase	0.97 (0.96–0.97)	NS	0.97 (0.95–0.98)	NS	0.97 (0.96–0.98)	1.06 (0.99–1.13)
Cholesterol (mg/dL)	10-unit increase	0.98 (0.97–0.98)	NS	0.97 (0.96–0.98)	0.94 (0.88–1.01)	0.97 (0.97–0.98)	0.96 (0.91–1.01)
Bilirubin (mg/dL)	0.1 unit increase	1.01 (1.01–1.02)	NS	NS	NS	NS	NS
% Bilirubin change	20-unit increase	1.01 (1.01–1.02)	1.08 (1.03–1.14)	NS	NS	1.03 (1.02–1.03)	NS
RBC (M/cmm)	1 unit increase	NS	NS	0.90 (0.84–0.97)	NS	0.93 (0.87–0.99)	NS
RBC change (M/cmm)	1 unit increase	NS	NS	NS	NS	0.72–(0.51–1.03)	NS

Abbreviations: NS, not selected in the multivariable model; Ref, reference level.

Fewer parameters were selected in the female cohorts, which were significantly smaller than the male cohorts (Table 3). Each year of increase in age was associated with 2% to 4% increased risk of PDAC. Other than age, no other variable was selected for in all three female cohorts, but several variables had individual significance in 1 or 2 cohorts. Of note, current NAFLD disease was associated with increased hazard of PDAC in cohorts (i) and (iii), as compared with no NAFLD disease.

### Model performance

**Figure 2.**
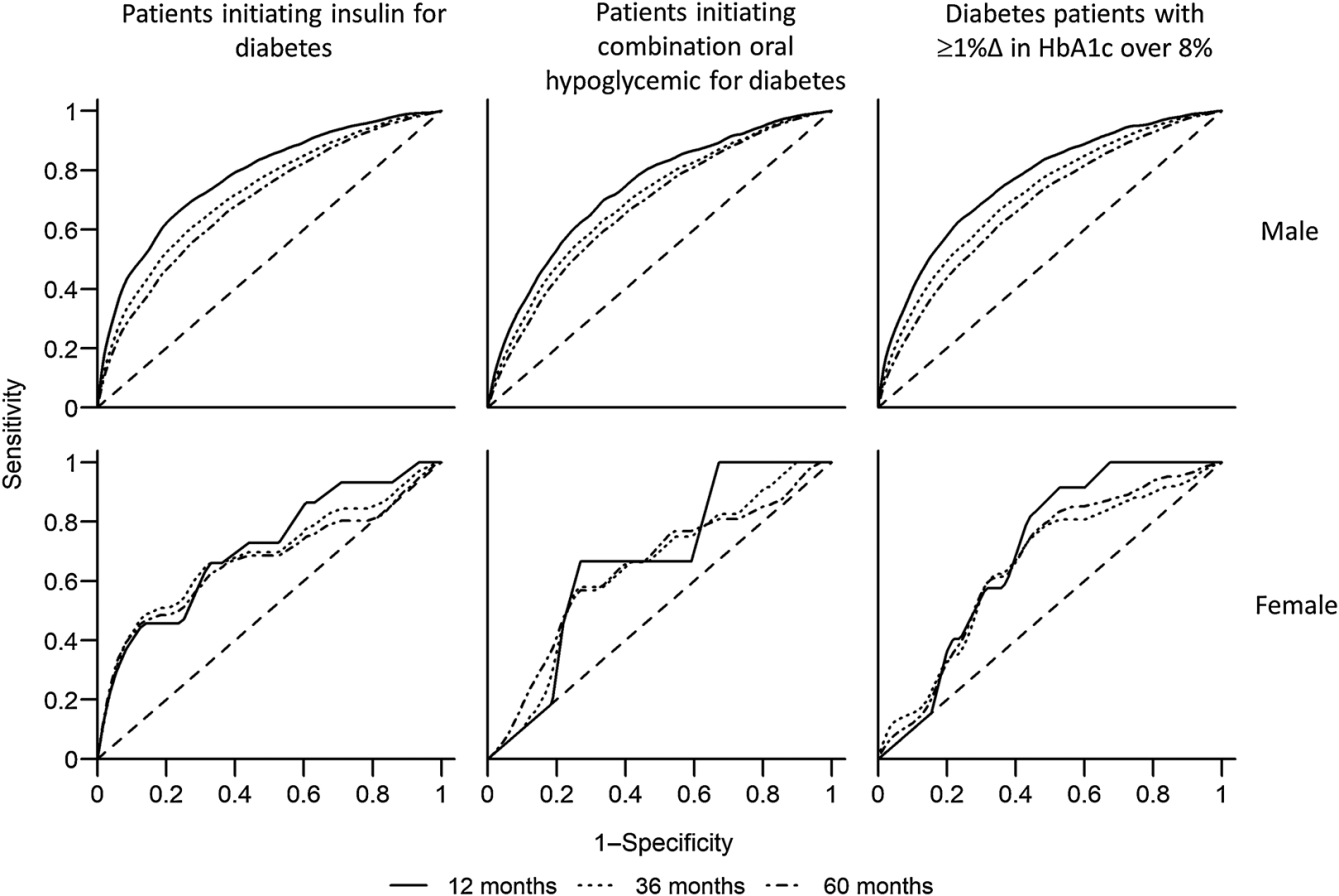
ROC for prediction of pancreatic cancer in veterans with progression of diabetes.

C-index values for the original data and the optimism-corrected values for each model are presented in Table 4. Corresponding receiver operator curves (ROC) of models of 12, 36, and 60 months risk of PDAC are presented in Fig. 2. Several trends are noticeable: (i) the models performed best for predicting the 12-month risk of PDAC (solid curve), as compared with predicting 36-month (dotted curve) or 60-month (dot–dashed curve) risk of PDAC; (ii) the models perform better for the insulin-initiating cohort and those with increasing HbA1c levels than for patients initiating combination oral hypoglycemic agents; (iii) the model performs better among males than in females. Among males initiating insulin, the optimism-corrected c-statistic for the 12-month incidence was 0.78, and for the 36-month incidence was 0.72. Among males with ≥1% increase in HbA1c over 8%, the model-predicted 12-month incident PDAC with *c* = 0.76 and 36-month incident PDAC with *c* = 0.71. Given that jaundice is a potential late-stage indicator of PDAC, we have performed sensitivity analysis excluding jaundice from the models for prediction of 12-month incidence of PDAC. No change in the performance of the models was noted, except among males with insulin initiation, in whom optimism-corrected c-statistic for the 12-month model decreased from 0.777 to 0.776. Among females initiating insulin, optimism-corrected c-statistic for the 12-month incident PDAC was 0.68, and for the 36-month incidence was *c* = 0.65. Among females with ≥1% increase in HbA1c over 8%, the model-predicted 12-month incident PDAC with *c* = 0.68 and 36-month incident PDAC with *c* = 0.63. Model performance among those initiating oral combination hypoglycemic treatment was lower than cohorts (i) and (iii) in both males and females. Optimism-corrected calibration slopes for prediction models ranged between 0.953 and 0.977 in males, and 0.802 and 0.870 in females.

**Table 4.** Summary of model performance for identifying future occurrence of pancreatic adenocarcinoma in veterans with progression of diabetes.

		Male		Female	
Cohort	Months	Original c-statistic (95% CI)	Optimism-corrected c-statistic (95% CI)	Original c-statistic (95% CI)	Optimism-corrected c-statistic (95% CI)
Patients initiating insulin for diabetes	12	0.779 (0.764–0.793)	0.777 (0.775–0.778)	0.710 (0.568–0.862)	0.680 (0.654–0.706)
	36	0.725 (0.712–0.740)	0.723 (0.721–0.724)	0.689 (0.559–0.812)	0.653 (0.633–0.682)
	60	0.695 (0.685–0.707)	0.693 (0.691–0.694)	0.612 (0.482–0.752)	0.595 (0.575–0.611)
Patients initiating combination oral hypoglycemic treatment for diabetes	12	0.736 (0.710–0.763)	0.733 (0.729–0.735)	0.667 (0.637–0.965)	0.653 (0.632–0.885)
	36	0.698 (0.683–0.717)	0.695 (0.691–0.696)	0.636 (0.452–0.824)	0.599 (0.578–0.627)
	60	0.676 (0.662–0.691)	0.672 (0.669–0.673)	0.641 (0.558–0.795)	0.592 (0.569–0.623)
Population with ≥1% increase in HbA1c over 8%	12	0.762 (0.748–0.780)	0.760 (0.758–0.761)	0.694 (0.589–0.810)	0.678 (0.653–0.693)
	36	0.715 (0.705–0.726)	0.712 (0.710–0.714)	0.651 (0.555–0.759)	0.632 (0.606–0.644)
	60	0.680 (0.673–0.692)	0.678 (0.676–0.679)	0.661 (0.586–0.742)	0.640 (0.622–0.653)

Model sensitivity, specificity, and PPV for predicting PDAC at ≥0.5%, ≥1%, and ≥2% predicted risk thresholds for the male diabetes populations are presented in Table 5. At a predicted PDAC risk threshold of ≥0.5% over 12 months, the PPV of the model for male insulin initiators was 1.41%, with a sensitivity of 35.7% and specificity of 94.3%. At a predicted risk threshold of ≥1% over 36 months, the PPV of the model for male insulin initiators is 1.89%, with a sensitivity of 26.4% and specificity of 94.1%. At a predicted risk threshold of ≥1% over 60 months, the PPV of the model for insulin initiation male population is 1.35%, with a sensitivity of 39.3% and specificity of 83.8%. At a predicted risk threshold of ≥1% PDAC risk over 36 months, the PPV of the model for oral hypoglycemic initiating male population reaches 2.2%, and that for male population showing increasing A1c reaches 1.77%. However, in both models the sensitivities fall below 20% with the predicted PDAC risk thresholds of ≥1% over 36 months. Accuracy measures and PPV were lower for the female diabetes populations (Supplementary Table S3).

**Table 5.** Sensitivity, specificity, and PPV of the model at various predicted thresholds in males with progression of diabetes.

Population	Incidence period	Predicted risk threshold	Sensitivity (95% CI) in %	Specificity (95% CI) in %	PPV (95% CI) in %	
Male patients initiating insulin	12 months	≥0.5%	35.68 (32.7–38.74)	94.31 (94.24–94.38)	1.41 (1.26–1.56)	
	12 months	≥1%	11.86 (9.92–14.03)	99.3 (99.28–99.33)	3.73 (3.1–4.45)	
	12 months	≥2%	4.82 (3.58–6.35)	99.88 (99.87–99.89)	8.39 (6.25–10.97)	
	36 months	≥0.5%	64.13 (61.91–66.3)	67.93 (67.79–68.07)	0.85 (0.8–0.9)	
	36 months	≥1%	26.44 (24.46–28.5)	94.1 (94.03–94.17)	1.89 (1.73–2.06)	
	36 months	≥2%	8.05 (6.86–9.37)	99.29 (99.27–99.32)	4.65 (3.95–5.43)	
	60 months	≥0.5%	79.23 (77.57–80.82)	42.77 (42.62–42.92)	0.77 (0.74–0.81)	
	60 months	≥1%	39.31 (37.37–41.27)	83.75 (83.64–83.86)	1.35 (1.26–1.43)	
	60 months	≥2%	12.52 (11.24–13.89)	97.76 (97.72–97.81)	3.06 (2.73–3.42)	
Male patients initiating oral hypoglycemia	12 months	≥0.5%	1.52 (0.56–3.28)	99.88 (99.87–99.89)	1.19 (0.44–2.57)	
	12 months	≥1%	0 (0–0.93)	99.99 (99.99–99.99)	0 (0–11.22)	
	12 months	≥2%	0 (0–0.93)	100 (100–100)	0 (0–52.18)	
	36 months	≥0.5%	21.65 (19.17–24.29)	93.24 (93.17–93.32)	0.81 (0.71–0.92)	
	36 months	≥1%	3.79 (2.71–5.14)	99.56 (99.54–99.58)	2.16 (1.54–2.95)	
	36 months	≥2%	0.58 (0.21–1.26)	99.97 (99.97–99.98)	5 (1.86–10.57)	
	60 months	≥0.5%	56.03 (53.54–58.5)	67.18 (67.04–67.33)	0.66 (0.62–0.71)	
	60 months	≥1%	10.6 (9.12–12.22)	96.47 (96.41–96.53)	1.16 (0.99–1.35)	
	60 months	≥2%	1.71 (1.13–2.48)	99.77 (99.76–99.79)	2.87 (1.9–4.15)	
Male patients with ΔHbA1c ≥1% with last HbA1c ≥8%	12 months	≥0.5%	17.36 (15.04–19.89)	98.1 (98.07–98.14)	1.53 (1.31–1.77)	
	12 months	≥1%	4.19 (3.02–5.64)	99.83 (99.82–99.84)	4 (2.88–5.38)	
	12 months	≥2%	1.33 (0.71–2.26)	99.98 (99.98–99.99)	11.5 (6.27–18.87)	
	36 months	≥0.5%	52.16 (49.99–54.32)	77.34 (77.23–77.45)	0.82 (0.78–0.88)	
	36 months	≥1%	14.91 (13.41–16.51)	97.02 (96.97–97.06)	1.77 (1.58–1.98)	
	36 months	≥2%	3.55 (2.8–4.43)	99.74 (99.73–99.75)	4.69 (3.7–5.85)	
	60 months	≥0.5%	72.69 (71–74.33)	51.33 (51.2–51.46)	0.72 (0.69–0.76)	
	60 months	≥1%	28.24 (26.58–29.94)	89.15 (89.07–89.23)	1.25 (1.17–1.34)	
	60 months	≥2%	6.4 (5.52–7.37)	98.93 (98.9–98.95)	2.83 (2.44–3.27)

## Discussion

We estimated the incidence of PDAC and developed and evaluated sex-specific models for prediction of PDAC in three populations with progression of diabetes in a nationwide sample of veterans. Cumulative incidence over 36 months from the time of progression of diabetes varied by definition of progression and by sex, with insulin-initiating males showing the highest incidence of PDAC diagnosis (0.37%). The models can predict the 12- and 36-month risk of PDAC with moderate accuracy among male veterans initiating insulin for diabetes and male veterans with increasing A1c levels. Male diabetes patients whose model-predicted 12-month risk of PDAC was ≥0.5% in these cohorts experienced actual PDAC incidence of 1.4%–1.5% over 12 months. This demonstrates that our models can identify high-risk patients in a substantial proportion of diabetes patients in whom earlier detection of pancreatic cancer may be feasible and warranted.

Beyond accuracy of prediction models, it is important to quantify the risk of PDAC in diabetes populations from various stages of diabetes for consideration of PDAC screening feasibility, risks, and benefits. The range of average annual incidence of PDAC observed in the male and female VA populations with insulin initiation (0.13%–0.18%) is substantially higher than that of the general adult population (20 per 100,000, or 0.02%) in the United States (38). The three-year risks estimated for insulin-dependent males and females (0.37% and 0.24%) are higher than estimated for veterans with new-onset diabetes defined by diagnostic codes (0.25%) and higher than the general veteran population (0.11%; ref. 39). The observed incidence in insulin initiators is similar to that of new-onset diabetes defined by ICD codes (0.4%; ref. 14) or by measures of glucose control (0.26%–0.50%; ref. 40), yet lower than new-onset diabetes with prior documented normal glucose (0.85%; ref. 41). Prediction model performance for 36-month incidence of PDAC in male insulin initiators (c-statistic = 0.72 for males) was lower than that estimated in the END-PAC model incorporating longitudinal data on weight and glucose control (c-statistic = 0.87; ref. 15), but comparable to the performance of the END-PAC model in an external population (c-statistic = 0.75; ref. 16) and comparable to the performance of a model among new-onset diabetes population in the UK (c-statistic = 0.81; ref. 14).

The pressing question of whether or not to screen for PDAC in patients with 1% to 2% model-predicted risk of PDAC over 1 to 3 years is a matter of weighing the costs, risks, and benefits of screening, which would generally involve magnetic resonance imaging or endoscopic ultrasound. Cost-analysis studies for screening diabetes patients for PDAC are under way and preliminary results point to potentially cost-effective strategies with <$100,000 per quality-adjusted life year gained (42). Screening for pancreatic cancer is now recommended for those with a family history of pancreatic cancer or individuals with high-risk germline mutations who face a lifetime PDAC risk of 4% to 40% (43). Annual surveillance by upper endoscopy is considered cost-effective for early detection of pancreatic cancer in these populations (44–46). Given the substantially high risk for PDAC among insulin-initiating populations with predicted probabilities ≥0.5% over 12 months and ≥1% over 36 months, screening may be cost-effective to implement at the time insulin initiation and further studies are warranted. Moreover, the model may help to improve the predictive performance of biomarkers by identifying high-risk individuals with higher prior probability of PDAC.

Our models identified consistent predictors of PDAC among male patients with progression of diabetes. These included current smoking, non-Hispanic ethnicity, acute pancreatitis, abdominal pain, jaundice, alcoholism, weight loss, increase in HbA1c, and lower levels of cholesterol. With the exception of Hispanic ethnicity, the identified risk factors have also been selected for in prior models of PDAC in diabetes patients (14, 15, 39). Our study of over 3,000 PDAC cases was well powered to detect these multiple predictors as independent risk factors of PDAC among males. Of note, weight loss and increasing glucose levels have received particular attention as potential early detection markers of PDAC (15, 16, 40, 47, 48). That these metabolic factors also predict PDAC in the three different stages of progression of diabetes confirms their predictive utility across the spectrum of diabetes. Novel factors identified in at least two of our models included Hispanic ethnicity, NAFLD, and change in bilirubin levels.

Our nationwide veteran male population consisted of a large number of Hispanic veterans, in whom the risk of PDAC was 21% to 24% lower as compared with non-Hispanic white patients in all three diabetes cohorts considered. Our observation is consistent with the ethnicity-specific trends reported among new-onset diabetes patients in an independent health system in Southern California (40). The mechanism by which Hispanic patients face lower risk of PDAC conditioning on diabetes status is unknown. Hispanic patients face higher risk for diabetes in part due to NAFLD (49). It is possible that liver fat-related metabolic dysfunction could be potential mediators of PDAC risk in the Hispanic population.

Epidemiologic data support the link between hepatic fat and PDAC. Pan-cancer studies comparing incidence rates of cancer in persons with and without NAFLD demonstrate that NAFLD is associated with increased risks of multiple cancer types, including PDAC (50–52). Epidemiologic investigations comparing liver fat content in PDAC cases and controls (53) also demonstrate positive association between NAFLD and PDAC. Our study demonstrates the temporal relationship between diagnosed NAFLD and PDAC in persons with progression of diabetes with fine control for other metabolic parameters, therefore suggesting a potential impact of organ-specific fat on pancreatic cancer, independent of glucose control and obesity.

A novel aspect of our model is the consideration of duration of binary risk factors. Prior studies have shown that recent use of PPIs (18), and recent development of pancreatitis (54) are associated with greater risk of PDAC than risk factors of more distant past. Our own analyses of Medicare demonstrate that several medical diagnoses are more frequently diagnosed closer to the onset of PDAC (55). Recent health changes due to the development of PDAC were evident in several risk factors we investigated: acute and chronic pancreatitis, abdominal pain, jaundice, DPP-IV inhibitor use, the recent onset of which was more strongly associated than more distant diagnosis/use. Of note, DPP-IV inhibitor has an immunomodulatory effect (56) and its use has been associated with PDAC in humans (57). Greater risk associated with more recent use, rather than more distant use, suggests a diabetes prescription change in response to suboptimal glucose control.

Despite many strengths, our study has a few limitations with regard to generalizability and outcome ascertainment. The female population comprised less than 10% of the veteran population we analyzed. Risk of cancer, other than breast cancer (58–62), is poorly understood in the female veteran population. Obesity and diabetes, which increase the risk of PDAC, are the most common metabolic conditions among female veterans who use the VA services (63). By year 2043, female veterans are projected to double (64), given broadening opportunities for women in the military. Although the prediction models for female veterans were limited in power, the PDAC risk and the associated risk factors we determined will have important implications for the millions of female veterans who will use VA services in the future. Our study was also limited in that a considerable proportion of patients were not identified through the tumor registry (21), but through coded diagnoses in the medical records or in Medicare claims. The use of at least two encounters with ICD diagnosis of PDAC increases the specificity of the case identification, and the use of Medicare claims overcomes the limitations of identifying PDAC cases occurring beyond VA services. If non-PDAC cases were counted as PDAC cases, this would have biased our incidence estimates higher, while potentially diluting the model performance. Lastly, the age-adjusted incidence of PDAC estimated for nonprogressing diabetes comparison group could have differed from the progressing diabetes population by factors unaccounted for.

In conclusion, we estimated 12- to 60-month risks of PDAC in men and women at different stages of diabetes progression and found that risk is substantially higher in the diabetes populations than in the general population, especially in those with insulin initiation. The prediction models reach moderate accuracy for identifying male population at high risk for PDAC, in whom surveillance studies may be warranted. External validation studies for evaluating the performance of our prediction models in an independent setting are needed.

## Authors' Disclosures

S. Kim reports grants from NIH/NCI during the conduct of the study. M.O. Goodarzi reports grants from NIH outside the submitted work. T.K. Nuckols reports grants from NIH/NCI during the conduct of the study. No disclosures were reported by the other authors.
